# Real-time active constraint generation and enforcement for surgical tools using 3D detection and localisation network

**DOI:** 10.3389/frobt.2024.1365632

**Published:** 2024-03-18

**Authors:** Spyridon Souipas, Anh Nguyen, Stephen G. Laws, Brian L. Davies, Ferdinando Rodriguez y Baena

**Affiliations:** ^1^ Mechatronics in Medicine, Imperial College London, Mechanical Engineering, London, United Kingdom; ^2^ Department of Computer Science, University of Liverpool, London, United Kingdom

**Keywords:** active constraints, surgical robotics, surgical tool detection, 3D pose estimation, surgical tool localisation

## Abstract

**Introduction:** Collaborative robots, designed to work alongside humans for manipulating end-effectors, greatly benefit from the implementation of active constraints. This process comprises the definition of a boundary, followed by the enforcement of some control algorithm when the robot tooltip interacts with the generated boundary. Contact with the constraint boundary is communicated to the human operator through various potential forms of feedback. In fields like surgical robotics, where patient safety is paramount, implementing active constraints can prevent the robot from interacting with portions of the patient anatomy that shouldn’t be operated on. Despite improvements in orthopaedic surgical robots, however, there exists a gap between bulky systems with haptic feedback capabilities and miniaturised systems that only allow for boundary control, where interaction with the active constraint boundary interrupts robot functions. Generally, active constraint generation relies on optical tracking systems and preoperative imaging techniques.

**Methods:** This paper presents a refined version of the Signature Robot, a three degrees-of-freedom, hands-on collaborative system for orthopaedic surgery. Additionally, it presents a method for generating and enforcing active constraints “on-the-fly” using our previously introduced monocular, RGB, camera-based network, SimPS-Net. The network was deployed in real-time for the purpose of boundary definition. This boundary was subsequently used for constraint enforcement testing. The robot was utilised to test two different active constraints: a safe region and a restricted region.

**Results:** The network success rate, defined as the ratio of correct over total object localisation results, was calculated to be 54.7% ± 5.2%. In the safe region case, haptic feedback resisted tooltip manipulation beyond the active constraint boundary, with a mean distance from the boundary of 2.70 mm ± 0.37 mm and a mean exit duration of 0.76 s ± 0.11 s. For the restricted-zone constraint, the operator was successfully prevented from penetrating the boundary in 100% of attempts.

**Discussion:** This paper showcases the viability of the proposed robotic platform and presents promising results of a versatile constraint generation and enforcement pipeline.

## 1 Introduction

The field of robotic surgery has enjoyed a steep increase in attention over the past years, both in terms of research, but also in terms of monetary investment. Robots have been developed for numerous types of surgeries, boasting different sizes, cutting capabilities, and overall increased accuracy compared to conventional methods (Walgrave et al., 2022). In orthopaedics, surgical robots have been reported to provide increased industry benefits across the majority of available literature for joint surgery (Elliott et al., 2021).

Surgical robots can be classified in categories depending on their operation and the level of surgeon involvement. Specifically, a robotic platform may be considered fully autonomous, thus operating under the surveillance of a surgeon, or collaborative, where the human operator actively manipulates the robot. The latter class of robotic systems requires the surgeon to manipulate the robot end-effector, which is usually a surgical tool itself, in order to perform precise operations on the patient anatomy. Collaborative robots may be further subdivided into teleoperated systems and hands-on systems (Sylvia, 2022). Teleoperated platforms comprise a master/slave configuration, where the robot can reproduce motions performed remotely by surgeons. Conversely, hands-on platforms require the surgeon to constantly manipulate the end-effector in space using their hand. This paper will primarily focus on *collaborative, hands-on* systems that require direct manipulation by the hands of surgeons. To better understand the field of robot assisted orthopaedic surgery, this section will initially provide a brief explanation of the general workflow undertaken during robotic surgery. Subsequently, the assistance offered by hands-on robots to surgeons, namely the concept of active constraints, will be analysed. This also involves an exploration of the predominant types of feedback provided by robotic systems to operators, which steered the development of the novel robotic system presented in this paper. Finally, a brief investigation of some commercially available hands-on robots for orthopaedic surgery will be provided, helping the reader obtain an understanding of parameters such as size, haptic capabilities, and other parameters that are also relevant to the novel robotic system presented in following sections.

In order to understand specific functions of a surgical robot, along with the benefits these functions offer to the surgeon in operation, it is important to first explore the workflow of a computer assisted orthopaedic surgery.

The first task is to achieve spatial registration between patient anatomy and robotic system prior to operation. More specifically, it is possible to “inform” the robot of the anatomy being operated by identifying points of interest, also called landmarks, across the operated tissue. This can be done in two ways, either through image-based techniques or imageless techniques (Ewurum et al., 2018). Image-based techniques, as the name suggests, rely on preoperatively obtained CT or MRI images of the operated anatomy. These images can be matched to the exposed anatomy by matching landmarks between the images and the tissue of the patient. The model of the anatomy can then be communicated to the robot, thus defining regions of interest in the workspace of the robotic platform. Conversely, imageless techniques require the identification of landmarks directly on the operated anatomy, without employing any images from the preoperative step. In this case, landmarks can be manually identified by the surgeon, and through a morphing process, the operated geometry can be entirely defined within the robot workspace (Innocenti and Bori, 2021). The preoperative plan also involves surgeon decisions such as selecting an appropriate position and dimensions of an implant. Table 1 demonstrates some commercially available orthopaedic surgical systems, along with the data required to achieve spatial registration.

**TABLE 1:** Orthopaedic Surgical Robots: Some of the currently commercially available surgical systems are listed on this table.

System	Company	Degrees-of-Freedom	Surgical procedure	Control method	Data requirements	Tracking method	Footprint (m^2^)
MAKO	Stryker	6	TKA, UKA, THA	Haptic	Image-based	Motor Encoders	>1
NAVIO	Smith & Nephew	N/A (handheld)	TKA, UKA	Boundary Control	Imageless	Optical Tracking	<0.5
CORI	Smith & Nephew	N/A (handheld)	TKA, UKA	Boundary Control	Imageless	Optical Tracking	<0.5
OMNIBotics	Corin Group	N/A (mounted on bone)	TKA	Cutting Guide	Imageless	Optical Tracking	<0.5
VELYS	DePuy Synthes	3	TKA	Haptic	Imageless	Motor Encoders	0.5–1
ROSA	Zimmer Biomet	6	TKA, THA, Spine	Cutting Guide	Both	Motor Encoders	>1

Note, TKA, Total Knee Arthroplasty; THA, Total Hip Arthroplasty; UKA, Unicondylar Knee Arthroplasty. This table was constructed by combining data from (Walgrave et al., 2022; Ewurum et al., 2018; Innocenti and Bori, 2021; Li et al., 2022; Chen et al., 2018). Note, footprint values refer to the mechanism dimensions exclusively.

Once registration has been achieved, the robot can be manipulated to operate on the patient. However, one major prerequisite of robot operation is the ability to track the end-effector in space. This can be achieved in different ways, but the two relevant techniques in orthopaedic computer assisted surgery are either the employment of tracking sensors, and specifically optical tracking methods (Ewurum et al., 2018), or the combination of optical tracking and robot motor encoders for actuated robotic platforms. An optical tracking system can be used for tracking non-actuated robotic platforms, registering preoperative images to the patient anatomy, registering the anatomy to the robot workspace, accounting for any dislocations of the operated tissue (e.g. knee flexion) during surgery, or performing initial registration of an actuated robot with respect to the patient anatomy. Optical trackers are highly accurate sensors, capable of tracking fiducial markers in space. However, since they are sometimes deployed in the operating room separately from the robotic device itself, they occupy additional operating room footprint. Tooltip tracking allows for the generation of visualisation platforms, which allow surgeons to visualise the location of the surgical tool with respect to the operated anatomy.

During robot manipulation of hands-on systems intraoperatively, the robotic platform can provide significant levels of support to the operator. One of the ways of assisting a surgeon is the concept of “active constraints.” This term can be used to describe control algorithms that provide some degree of assistance to the human operator during robotic manipulation. Specifically, the process of establishing and applying an active constraint comprises two steps, namely the constraint definition and the constraint enforcement. Active constraint definition is the process of generating a geometry with a fully defined boundary in the workspace of a robot. Once the constraint geometry has successfully been established in the robot workspace, it is enforced upon the robot. This is achieved by identifying the configuration of the robot end-effector with respect to the constraint boundary and subsequently influencing the motion of the human operator (Bowyer et al., 2014), or generating other robot responses, such as speed reduction or even deactivation. Note that throughout this paper, the terms active constraint definition and boundary generation may be used interchangeably for describing the process of establishing the constraint geometry.

The process of constraint definition is usually achieved alongside the process of registering the patient anatomy to the robot workspace. For example, it is possible to utilise a point-cloud of the patient anatomy, match it to the preoperative images and ultimately register it to the robot. This allows for complete active constraint definition in robot coordinates. One example has been demonstrated with the use of a kidney point-cloud (Kastritsi et al., 2019). Subsequently, tracking allows for the calculation of the robot end-effector position with respect to the constraint boundary, thus allowing for active constraint enforcement upon interaction between robot tooltip and boundary.

Active constraint enforcement can usually be implemented through haptic feedback. In such cases, a physical reaction from the robot is communicated to the operator. Haptic feedback may be categorised into two groups. The first haptic feedback category is force feedback, which involves the application of a force from the robotic platform to the operator. The second category is tactile feedback, most notably implemented in the form of vibrations produced by the robot and transmitted to the operator’s hand. Of the two categories, force feedback has received significantly more attention (Okamura, 2009). Some developments can also be noted in visual feedback techniques, where the operator is able to better visualise the position of the robot with respect to either the patient tissue or the aforementioned predefined boundary through external monitors and augmented reality systems. External monitors may allow for the visualisation of important parameters, such as the force that a haptic system would theoretically apply to the operator or other visual clues (Hagen et al., 2008). Similarly, augmented reality solutions allow for the superimposition of virtual objects on the operated patient tissue (Seetohul et al., 2023), thus allowing for more detailed visualisations of the operated anatomy.

Substantial research has been undertaken on the topic of active constraints, and a general summary is available (Bowyer et al., 2014). Examining the process of active constraint definition, it is generally assumed that the constraint geometry has been defined *a priori*, or can be defined using some generated point-cloud (Sharp and Pryor, 2021; Kastritsi et al., 2019). Another frequent implementation involves the generation and combination of primitive shapes (e.g. spheres, cylinders etc.). These primitive shapes can then be combined to generate a more complex active constraint geometry (Bettini et al., 2004). It should be noted, however, that when exploring the concept of active constraint definition, generating the constraint geometry alone is not sufficient. The generated active constraint boundary needs to be “anchored” to the operated patient tissue. In doing so, any registered robotic system becomes capable to localise the generated geometry.

When it comes to constraint enforcement, numerous techniques have been developed, and a thorough exploration of these techniques is beyond the scope of this paper. However, two very relevant examples should be demonstrated, namely the enforcement of haptic force feedback across “safe-zones” and “restricted-zones.” In the former case, the operator may manipulate the robotic end-effector within the constraint boundary using their hand unperturbed, until an attempt to penetrate the boundary occurs. Upon boundary penetration, the active constraint is enforced through the robot by applying a restrictive force to the operator’s hand, thus hindering further deviation from the constraint boundary (Marques Marinho et al., 2019). Conversely, in the case of a restricted-zone, also referred to as a “forbidden region virtual fixture” (Abbott and Okamura, 2003), the operator experiences a repulsive force by the robot when the end-effector is approaching the active constraint boundary, thus limiting or entirely preventing constraint boundary penetration from the outside (Marques Marinho et al., 2019). A final distinction to make concerns dynamic environments (Ren et al., 2008). Static constraints assume that changes in the patient tissue (e.g. deformation of soft tissue) do not affect the active constraint boundary in operation. A dynamic constraint, however, takes into account patient tissue changes, thus updating the constraint geometry according to these alterations. Therefore, with the enforcement of an active constraint along the workflow of robotic surgery, robots possess an inherently higher level of safety upon interaction with human tissue, while also reducing the mental load experienced by a surgeon during operation (Marques Marinho et al., 2019). It should be noted that in active constraint enforcement via haptic force feedback, it is the motors incorporated across the robot that generate the force imposed on the operator. Hence, in some cases, the forces experienced by the surgeon can be tuned to allow the surgeon to consciously overcome the resistance imposed by the robotic platform and continue manipulating the end-effector beyond the constraint geometry.

With the enforcement of an active constraint depending on the motors used in a robotic system, it is essential to incorporate motors that are strong enough to overcome the forces that a surgeon would experience during operation. For example, in the case of bone grinding of a porcine skull, the forces experienced by the operator were shown to be in the range of 14N–23N (Babbar et al., 2020). In the case of drilling, wider ranges have been reported, from 25N and up to 75N in bovine (Waqas, 2015), which have been tested more, and 176N–198N for human femoral shafts (MacAvelia et al., 2012). For haptic feedback to be effective, the operator needs to be able to distinguish the forces produced by the robot for haptic force feedback from the forces experienced when operating on patient tissue. This poses no problem for robotic platforms with high operating room footprint, such as the MAKO system (Stryker, USA) (Martin, 2021), with the robotic device alone occupying 1.1 m^2^ (Synthes, 2021). In such cases, the motors required to overcome the bone drilling forces can be integrated to the design without affecting the overall footprint of the system significantly. However, in the cases of miniaturised robots, heavier motors may pose a problem in the ease of end-effector manipulation, as the load imposed on the operator can increase with robot device weight. This means that miniaturised systems may not be as able to provide haptic force feedback as bigger systems for applications such as bone drilling or grinding without significantly increasing the physical effort required by the operator.

Having understood the process of deploying a collaborative surgical robot in the operating room and registering it to the patient, as well as the process of imposing an active constraint, it is also important to understand the accuracy achieved by such systems. These values can be used as a reference point from any novel robotic platforms. In the sector of orthoapedics, collaborative, hands-on surgical robotic platforms deployed for cutting, as opposed to robots that support jig placement, can be arranged into two categories, depending on the type of support the robot provides to the operator who is manipulating the robot using their hands. These categories are haptic robots, and boundary control robots. The function of haptic feedback has been outlined in this section. Boundary control robots are similar to haptic feedback systems in the sense that an active constraint boundary across the patient geometry is established. However, instead of the robot providing haptic feedback to the operator upon interaction with the boundary, the robot instead partially or completely interrupts the function of the surgical tool used for the operation upon interaction with the boundary.

An example of haptic robots is the MAKO (Stryker, USA) (Martin, 2021), which is a six degrees-of-freedom system, used for unicompartmental knee arthroplasty (UKA), total knee arthroplasty (TKA), and total hip arthroplasty (THA). A surgeon manipulates surgical tools mounted on a robot arm, with the robot itself applying a restrictive force upon potential deviations from the constraint geometry. Studies have demonstrated that MAKO offers a significant degree of improvement across surgeries. In a cadaveric study of TKA, the femoral anterior, distal, and posterior flexion values were found to be 0.4°  0.8°  and 0.5° respectively, with the anterior and posterior flexion values demonstrating a significant reduction compared to the manual operation results of 4.7° and 2.3° respectively (Hampp et al., 2019).

Conversely, in the case of boundary control, a surgeon is once again required to control a robot end-effector, with cutting being prevented or entirely terminated upon exiting from the active constraint boundary. One such case is the NAVIO (Smith & Nephew, UK), used in TKA (Wu et al., 2021). This is a handheld tool that can either limit the speed of the surgical burr tooltip, or completely block the burr itself upon penetrating the boundary (Mancino et al., 2022). Cadaveric study results on TKA for the NAVIO demonstrated a mean femoral flexion of 2.0° (Casper et al., 2018), which is an improvement compared to standard methods. Similarly, the NAVIO achieved a femoral flexion of 1.23° in a cadaveric study for UKA (Khare et al., 2018).

A complete analysis of the progress of commercially available collaborative, hands-on surgical robots is beyond the scope of this paper. However, by examining Table 1, a pattern emerges. In particular, there is a trade-off between the haptic capabilities of a robotic system against its footprint in the operating theatre. On one side, there are systems like the NAVIO and its successor, the CORI (Smith & Nephew, UK). These platforms are miniaturised, handheld, and usually occupy less than 0.5 m^2^ in the operating room (Walgrave et al., 2022), while capable of providing boundary control. On the other side stand the bulkier systems, which allow for haptic feedback supported motion along fewer degrees-of-freedom, such as the MAKO. The observed pattern suggests that haptic force feedback can be more robustly supported by bigger robotic platforms, while smaller systems provide boundary control solutions. It should also be noted that some systems, like the ROSA platform, function as guides, meaning that they allow for precise placement of jigs.

This piece of research aims to address some of the points presented in this section. Specifically, the first point to address is that miniaturised systems do not provide haptic feedback, instead opting for boundary control solutions in the active constraint enforcement step. Furthermore, the process of active constraint definition involves the use of optical tracking methods and preoperative imaging techniques to fully define and anchor a constraint geometry on to the patient anatomy. Some applications also require the construction of point-clouds based on the preoperative imaging process, which also consumes time and may require surgeon supervision. With these two limitations in mind, this paper provides a twofold solution.

Firstly, a refined version of a collaborative, hands-on, three degrees-of-freedom robot, the Signature Robot is presented. This version is an improvement of the previously presented version (Souipas et al., 2022). This robotic platform addresses the chasm between bulky robots that offer haptic feedback and miniaturised systems that solely depend on boundary control by introducing a miniaturised system that is capable of haptic force feedback.

Secondly, an active constraint pipeline is introduced. This pipeline allows operators to define and enforce an active constraint “on-the-fly,” without the need for any preoperative imaging information or optical tracking techniques to anchor the constraint boundary on the patient tissue. Specifically, by using a surgical scalpel, the operator can intraoperatively define a static active constraint geometry. This is achieved by employing our previously presented SimPS-Net (Souipas et al., 2023a), a monocular, RGB (Red-Green-Blue), camera-based network capable of detection and 3D localisation of surgical tools. Through the use of this tracking network, the operator can define constraint geometries by manually moving the scalpel in 3D space, with its trajectory being recorded by the network to generate the active constraint boundary. This technique eliminates the need for an optical tracker intraoperatively for the purpose of active constraint definition. The generated constraint geometry can be subsequently used by the Signature Robot, which can enforce the constraint via haptic force feedback, thus allowing for a comparison between manual operation and operation under haptic feedback.

## 2 Methodology

The aims of this research paper can be divided into three sections. First, an exploration of haptic constraint capabilities of the Signature Robot, along with a plastic bone cutting study. The second aim is the achievement of an “on-the-fly” active constraint definition procedure. Lastly, the final purpose is the enforcement of the active constraint through the Signature Robot.

### 2.1 Signature Robot

**FIGURE 1:**
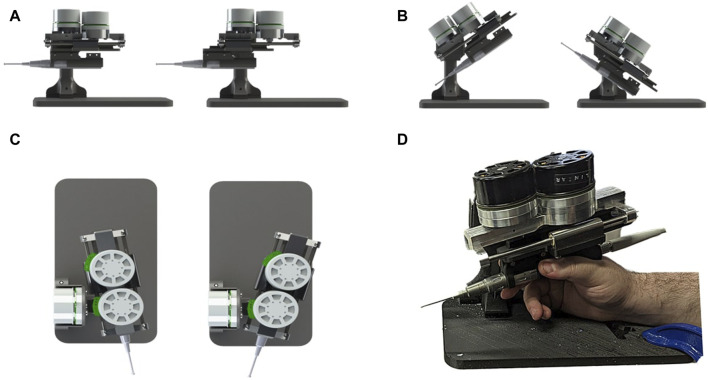
Demonstration of Signature Robot: Illustrates the motion capabilities of Signature robot in **(A)** linear translation, **(B)** pitch rotation and **(C)** yaw rotation. The system being manipulated by a human user is shown in **(D)**.

The development of the Signature Robot came about as a successor to the Acrobot (Jakopec et al., 2003). The purpose of this novel platform is to address the gap between bulky robots with haptic feedback capabilities and miniaturised, boundary control robots in orthopaedic surgery. With that in mind, the Signature Robot was formulated as a collaborative, hands-on system, allowing a human operator to manipulate the robot arm using their hand. Motion is achieved across three degrees-of-freedom, namely linear, yaw, and pitch, as shown in Figure 1. This design allows for operators to manipulate a surgical tool mounted on the robot arm using precise finger motions or minor wrist motions. The range of motion is 90 mm in linear translation, 65° in pitch and 40° in yaw. In the presented version, the robot arm is mounted with a MCI-270 surgical burr (deSoutter, UK), which the operator can manipulate with their hand to perform bone cutting and grinding operations.

The Signature Robot is a miniaturised platform, with the robotic device mechanism requiring 0.2 m^2^ to be deployed, including external components (e.g. power supply, controllers, etc.). In addition, it weighs 1.9 kg, making it easy to move in the operating room. Furthermore, it provides haptic force feedback to the operator. It is fitted with three Maxon EC 60 Flat motors, each directly controlling one degree-of-freedom. Each motor weighs 470g. The direct drive is an improvement from the previous version (Souipas et al., 2022), which suffered from mechanical backlash due to the use of transmission elements, such as bevel and spur gears. The motors in the presented version are capable of producing 401 mNm, which can be translated to a direct force of 100.3N in pitch and yaw. The linear force diminishes to 64.2N due to the incorporation of a rack and pinion mechanism for translational motion. These forces exceed the range force involved in bone burring reported in Section 1. Therefore, active constraint enforcement can be effectively achieved in bone cutting operations. For the purpose of motor control, the motors are each connected to a EPOS4 Compact 50/15 EtherCAT controller. The controllers are then connected via ethernet to an embedded computer, Esmacat Master S, thus allowing for the reading motor values and implementing of constraints. This setup allows for data readings at a rate of 500fps. The computer is also connected to an external monitor, thus allowing for visualisations that will be explored later.

Similarly to the preceding version (Souipas et al., 2022), this version of the Signature Robot allows the operator to define and enforce an active constraint. In one active constraint implementation, constraint definition can be performed by employing primitive shapes, such as a sphere or a cylinder. The operator may choose the location to place this constraint. Subsequently, the operator may use their hand to freely manipulate the end-effector within the active constraint geometry, but upon penetrating the constraint boundary, the active constraint is enforced through a viscoelastic force, calculated in Eq. 1:

$$F=k\cdot d\cdot \hat{p}+c\cdot \dot{x}$$ (1)

where:

**F** = force vector on the robot tooltip.

**k** = [*k*_*x*_, *k*_*y*_, *k*_*z*_], the elastic constants vector.

*d* = robot end-effector distance from the active constraint boundary.

$\hat{p}$ = unit penetration vector.

**c** = [*c*_*x*_, *c*_*y*_, *c*_*z*_], the damping coefficients vector.

$\dot{x}$ = robot velocity vector.

The above equation demonstrates a translational force vector which is applied by the motors to the hand of the operator upon penetrating the constraint boundary. Specifically, this restrictive force hinders further constraint penetration, which increases in magnitude the further away the tooltip is from the constraint boundary. It should be noted, however, that should the operator consciously decide to further penetrate the boundary, the restrictive force can be overcome, especially at small penetration distances. As a result, this allows an operator to remain in control, without the robot entirely preventing movements that they may perform.

In Equation 1, the elastic constant vector, **k**, and the damping constant vector, **c** were empirically set to **k** = [2.5, 2.3, 2.0]*kN* ⋅ *m* ^−1^ and **c** = [0.3, 0.3, 0.2]*kN* ⋅ *s* ⋅ *m* ^−1^ respectively. Both these vectors depend on the application performed using the robot, as well as operator preference. Higher values of **k** will lead to a stiffer constraint, thus minimising distance from boundary upon exit. However, this could potentially prevent the surgeon from consciously penetrating the boundary. Higher damping values, on the other hand, restrict high speed motions. Furthermore, $\hat{p}$ is the unit vector between the current robot position and the constraint geometry centre or central axis. For example, in the case of a cylindrical constraint geometry, the force will always point towards the cylinder axis. With Eq. 1 responsible for calculating the translational forces that the robot must apply to the operator, Eq. 2 can be used to determine the motor torques that must be generated in each joint to produce the calculated force.

$$\tau =J^{T}\left(q\right)\cdot F$$ (2)

where:

***τ*** = vector of required motor torques.

***J*** = Jacobian matrix.

***q*** = joint angle vector.

***F*** = vector of desired forces at the robot tooltip.

The Jacobian matrix can be calculated through robot inverse kinematics, thus allowing for the calculation of ***τ***. Note that this equation has been simplified from its complete version (Caccavale et al., 1997), based on the assumptions that in operation, the robot tooltip is manipulated at low speed and also that the operator is absorbing the entirety of the system weight by holding the robot arm, thus minimising gravity and inertia effects. It should be noted that Eq. 1 demonstrates that restrictive force rises the further away the operator moves from the constraint boundary. This force will keep increasing as the distance rises, until the maximum force that can be exhibited by each motor is reached. At that point, the motors may not exhibit a higher force, so a plateau is achieved. However, it is highly unlikely that an operator will be able to overcome this value exclusively via finger motions.

**FIGURE 2:**
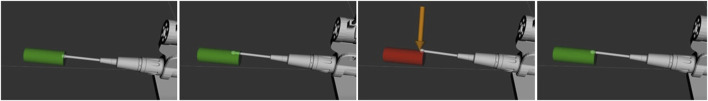
Demonstration of Active Constraint on Signature Robot: Demonstrates unrestrained manipulation of the surgical burr while within the cylindrical constraint, with no forces acting on the tooltip and the cylinder being green. Upon constraint penetration, the cylinder turns red and a restrictive force is applied to gently guide the human user back within the constraint volume.

Alongside the implementation of haptic force feedback across the workflow of the Signature Robot, visual feedback was also implemented. Specifically, a digital twin was developed from the kinematic model of the robot. The digital twin mimics the motions of the physical system by reading the rotations of the motors, thus allowing for visualisation of the system motions in a simulation space. The active constraint implementation may also be visualised in the same space, as demonstrated in Figure 2. As observed, the constraint geometry, defined as a cylinder, has been adapted to provide visual feedback. Specifically, colour changes are incorporated. Motion within the constraint geometry is shown in green, where the operator can manipulate the end-effector unrestricted. However, upon penetrating the active constraint boundary, the cylindrical geometry becomes red, and the force vector is also displayed. This is the visual feedback that can be provided to the operator during use.

A final point to make regarding the robotic platform concerns the positional accuracy of the system. A kinematic calibration process was undertaken in order to optimise the kinematic model parameters of the robot and also obtain the positional accuracy of the system. The method was similar to the previous design (Sciavicco and Siciliano, 2001), and the positional error of the robot, as well as the individual errors across each direction of motion, are listed in Table 2.

**TABLE 2:** Table of Average Accuracy of the Robot: Lists the Root Mean Square Error (RMSE) along all three directions of motion (± standard deviation) and also the average of these three individual values (± standard deviation), which is the total accuracy of the platform following kinematic calibration.

Average RMSE (mm)	RMSE-X (mm)	RMSE-Y (mm)	RMSE-Z (mm)
0.64 ± 0.41	0.58 ± 0.32	0.47 ± 0.16	0.46 ± 0.24

With both the design and the active constraint capabilities of the system having been established, a study to explore different modes of robot operation was undertaken.

### 2.2 Signature Robot plastic bone testing

**FIGURE 3:**
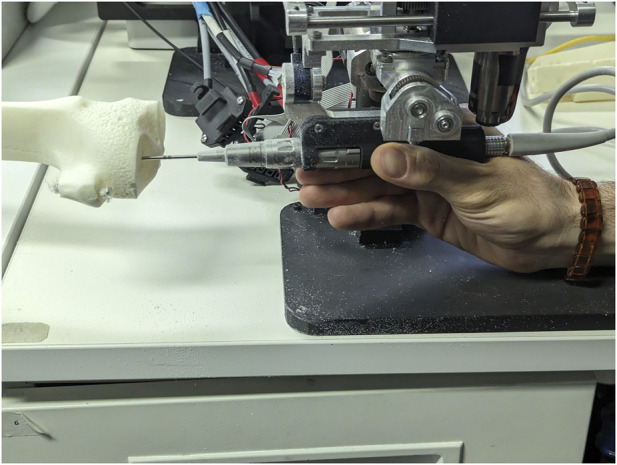
Experimental Setup for Plastic Knee Cutting: Illustrates how one of the volunteers held the surgical burr and interacted with a plastic bone specimen during testing.

In order to compare different modes of operation of the robot, 4 tests were undertaken, each with a different volunteer. The volunteers were all in the age range of 24–35, with a background in robotics. However, none of the volunteers had any prior experience with hands-on collaborative robot manipulation, with the only relevant experience being their previous use of a teleoperated system for laparoscopic surgery on a research level, not a professional one. In addition, none of the volunteers had been previously introduced to the Signature Robot system. The reason for choosing these volunteers was because they qualified as “untrained” with the examined robotic platform, while still being able to provide useful feedback regarding potential alterations in the design of the system. The robot was placed within range of a fixed plastic bone phantom, and the embedded computer monitor was placed near the robot. As mentioned, the robot was mounted with a MCI-270 surgical burr (deSoutter, UK) of 5 mm diameter. Figure 3 demonstrates the experimental setup, with the computer monitor not shown.

Regarding the bone phantom samples, they are made of a Plastic Cortical Shell. This material correctly mimics the external and internal structure of a bone, by offering a rigid exterior which encloses the trabecular bone phantom (Sawbones, 2019). Specimens like these are usually incorporated in the training of surgeons, or for experiments that aim to quantify performance across different operators of surgical systems. Despite not mimicking material properties of human bones, these samples allow for experiments that involve the exploration of robot manoeuvres in operation. An additional benefit is that no ethics approval is required to obtain such samples, since no human tissue is involved, thus allowing for more repetitions to be undertaken with ease. The discrepancy between real bone tissue properties and phantom properties is of no impact in this study, which evaluates the relative improvements under different forms of feedback.

Each test involved the cutting of a cylindrical section of 15 mm diameter on plastic bone using the Signature Robot under four different modes of operation. The tested modes of operation were freehand, visual feedback, haptic feedback, and combined feedback operation.

For each mode of operation, each volunteer was requested to define the starting point of a cylindrical active constraint geometry on the plastic bone and then proceed with the cut. The requirements for establishing the cylindrical volume were to ensure that the starting point of the cylinder was placed on the surface of the plastic bone specimen, whilst also ensuring the axis of the cylinder would be confound within the phantom body. This ensured that in no experiment did the volunteers end up manipulating the robot end-effector outside the plastic bone specimens. The parameters of the cylindrical constraint geometry were stored. Upon completing a duration of 120s, the trajectory of the robot during testing was also stored, along with the recorded timestamps, and the operator repeated the process for all four modes of operation. In freehand operation, no form of feedback was available. In visual feedback operation, operators had access to the digital twin monitor, thus receiving visual feedback identical to the one of Figure 2. In the third mode, haptic feedback was exclusively present, with the final mode of operation combining visual and haptic feedback.

Ultimately, each mode of operation was tested once from each volunteer. The data processing performed after collecting all required data was undertaken for each mode of operation individually, in order to obtain individual exit metrics for each mode, which could then be combined and compared with other modes of operation. The metrics obtained can be divided into two parts, namely boundary exit related metrics and volume removal metrics. Note, since the process of examining individual exits is the same in all four modes of operation, the process of obtaining both exit and volumetric metrics can be outlined for one mode of operation.

Boundary exit metrics explore individual exit patterns noted in each mode of operation. Specifically, an “exit” or “penetration” in this test is defined as any penetration of the active constraint cylindrical boundary. For each exit, the average individual exit distance and the maximum individual exit distance may be calculated as shown in Eqs 3, 4 respectively. Therefore, each mode of operation under a single volunteer may yield as many numerical results as the number of boundary penetrations.

$$D_{\text{exit}}=\frac{1}{n}\overset{n}{\underset{i=1}{\sum }}d_{i}$$ (3)

$$D_{\text{max, exit}}=\max\left(d_{1},d_{2},\ldots ,d_{n}\right)$$ (4)

where:

*D*_exit_ = average distance noted during each individual exit.

*D*_max, exit_ = maximum distance noted during each individual exit

*n* = number of measurements in the individual exit.

*d*_*i*_ = robot end-effector distance from constraint boundary at each measurement.

For each of the four modes of operation, the amalgamated metrics can be calculated by utilising the individual mean and maximum distance values. The *average individual exit distance* and the *average of maximum exit distances* are calculated according to Eqs 5, 6 respectively. Note, these metrics are obtained for a single mode of operation across all volunteers.

$${\bar{D}}_{\text{overall}}=\frac{1}{N}\overset{N}{\underset{i=1}{\sum }}D_{\text{exit},i}$$ (5)

$${\bar{D}}_{\text{max}}=\frac{1}{N}\overset{N}{\underset{i=1}{\sum }}D_{\text{max, exit},i}$$ (6)

where:

${\bar{D}}_{\text{overall}}$ = mean of individual average exit distances in a single mode of operation.

${\bar{D}}_{\text{max}}$ = mean of individual maximum exit distances in a single mode of operation.

*N* = total number of exits in a single mode of operation.

An identical method is followed to obtain metrics relevant to the duration of the exits noted during testing. Specifically, the *average individual exit duration* demonstrates the mean duration of an exit of the tooltip from the constraint boundary. Similarly, the *average of maximum exit durations* demonstrates the mean across the maximum duration values noted when testing a mode of operation across all volunteers.

Volume removal metrics, on the other hand, demonstrate the amount of volume removed from the plastic bone. Similarly to the exit metrics, the volumetric measurements address each mode of operation individually by amalgamating the results obtained across all volunteers.

Volumetric results address two parameters, namely the volume of plastic bone removed from within the active constraint geometry, and the total volume of plastic bone removed during testing. To measure the volume burred during each mode of operation for each volunteer, the robot end-effector position was used. A grid space was generated based on the maximum and minimum coordinates that the robot reached during testing. The grid points were set to have a fixed distance of 0.5 mm. When iterating across the recorded trajectory of the end-effector, the nearest grid point to the robot tooltip was located in the grid space and defined as “cut”. In addition, all grid points within the radius of the bur were also defined as removed volume. By iterating across the entire recorded trajectory in one mode of operation for a single volunteer, all removed grid points could be identified. Ultimately, the total volume removed in operation was calculated by approximating the volume of each grid point to a cube, based on the distance between points.

**FIGURE 4:**
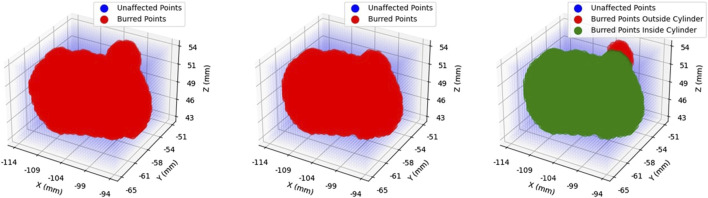
Volume Ratio Visualisation: Demonstrates the total volume removed in operation (left), the portion of that volume contained within the constraint region, namely the “inner” volume (middle), and the superposition of the two (right). Removed volumes are illustrated in red.

With the total volume obtained, and information regarding the cylindrical constraint geometry parameters available, it was then possible to extract the volume contained within the constraint geometry from the total volume. For one of the participants operating the robot in haptic feedback mode, Figure 4 shows the total volume burred and the volume burred within the active constraint. The red points indicate grid marks that the burr tooltip removed from the plastic bone during cutting. These two volumes are superimposed in the same figure to better visualise the ratio of “inner/total volume ratio,” which denotes the ratio of volume within the constraint (green) over the total volume.

Upon completing the experiment for all four modes of operation, volunteers were asked to also complete a NASA TLX assessment (Hart et al., 1998). This qualitative test was performed to further understand the perceived workload of users whilst operating the Signature Robot. The purpose of these assessments was to identify and potentially improve sources of discomfort across the design of the system. The assessment form was focused on the combined feedback mode of operation, since this mode is similar to the conditions a surgeon would experience when using the Signature Robot in the operating theatre. Since this is the most realistic mode of operation, the assessment forms tackled these conditions, and were ultimately averaged to locate potential shortcomings when operating the robotic platform. Note, the NASA TLX values may range from 0 to 20. Higher reported values typically indicate greater perceived workload or task difficulty across the majority of the tested parameters. While this applies to most of the metrics, such as “Mental Demand” and “Physical Demand”, the “Performance” metric evaluates the self-reported performance level on the task. When participants rate performance lower, it suggests that they perceive the task as more challenging.

Having established the operation of the Signature Robot and tested the haptic capabilities of the system, the first aim of this research, namely the construction of a collaborative, hands-on robot with haptic feedback, has been realised. With the completion of this robotic platform, the next step is to achieve the aim of first defining an active constraint, and subsequently enforcing is using the Signature Robot. In order to achieve active constraint definition, however, one prerequisite is the ability to track and localise objects in 3D space. To that end, it is first important to explore the network employed for the purpose of detection and tracking of surgical tools in operation.

### 2.3 SimPS-Net: network architecture

With the robot platform capable of providing haptic feedback examined in the previous subsection, the next requirement in this piece of research is the active constraint definition. A prerequisite of defining the active constraint geometry is to understand the network employed for tracking purposes. Specifically, one of the novelties of this research is the active constraint definition being achieved by tracking a standard surgical tool in 3D space, instead of utilising preoperative imaging. The operator may move this tracked tool, in the case of these experiments a scalpel, using their hand. The employed network, SimPS-Net  is used to track the scalpel in space, providing the 3D position and orientation of the surgical tool (Souipas et al., 2023b). Having recorded the position of the scalpel tip in space, it is possible to subsequently use this trajectory to construct the active constraint geometry, as will be outlined in Section 2.4.

**FIGURE 5:**
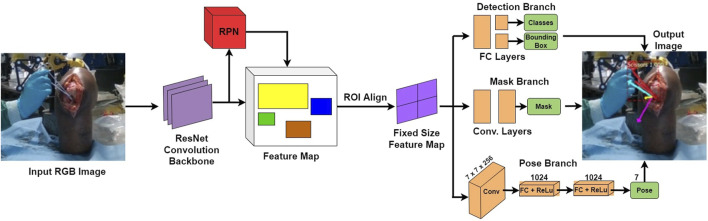
SimPS-Net Architecture: Demonstrates the architecture of the network developed for simultaneous detection and 3D pose estimation of surgical tools. The network receives a single, RGB image as an input and provides a segmented image along with the corresponding 3D pose of the detected tool within the frame.

The network SimPS-Net is capable of detecting a surgical tool in an image, but also inferring the 3D pose of the detected tool in camera 3D coordinates. It is an expansion of Mask-RCNN (He et al., 2017), a region-based network used for semantic object segmentation in images. The architecture of SimPS-Net is demonstrated in Figure 5. The original Mask-RCNN comprises the backbone, the detection branch and the segmentation branch. SimPS-Net introduces the pose branch, which allows for 3D pose regression of any tool detected by the previous two branches.

The input to this network is a single RGB image, thus making SimPS-Net a monocular, RGB, camera-based network. The pose branch initially applies a convolution step to the input image. The convolution step also involves a 7 × 7 pooling process, which is capable of extracting features from the input image. Upon extracting the necessary features, two fully-connected layer operations are applied to the output of the convolution step. This allows for further refinement of the extracted features, thus leading to a better understanding of the position and orientation of the surgical tool detected within the input frame. Finally, a dense, Rectified Linear Unit (ReLU) activation layer is applied to the output of the second fully-connected layer. This allows the pose branch to express the output obtained by processing the input image as a function of seven parameters. These seven parameters include three position and four orientation values, the latter being in quaternion format. The output 3D pose, **p**, is illustrated in Eq. 7:

$$p=\left[x,\;\theta \right]$$ (7)

where:

**x** = [*x*, *y*, *z*] is the position vector.

***θ*** = [*q*_*x*_, *q*_*y*_, *q*_*z*_, *q*_*w*_] is the orientation quaternion vector.

The pose branch is capable of inferring the 3D pose of a surgical tool detected in the input image, **p**_**pred**_. In order to optimise the network during training, the final step of the pose branch requires the solution of a pose loss function. The loss function of SimPS-Net is demonstrated in Eq. 8, where position and orientation are initially tackled independently, with their combination constituting the entire pose loss. Note that the constants *α*, *β* account for any scale difference between the position and orientation, while also improving the network results (Kendall et al., 2015).

$$L=\alpha \;{\left\|x_{\mathrm{true}},\;x_{\mathrm{pred}}\right\|}_{2}+\beta \;{\left\|\theta_{\mathrm{true}},\;\theta_{\mathrm{pred}}\right\|}_{2}$$ (8)

Even though accuracy results have been previously published, with the network achieving a mean position and orientation error of 5.5 mm ± 6.6 mm and 3.3° ± 3.1° respectively (Souipas et al., 2023a), the experiment presented in this paper is the first attempt at deploying the network in real-time. Therefore, to quantify the real-time inference success of the network, the success rate metric is employed, defined as shown in Eq. 9:

$$\text{success rate}=\frac{N_{\mathrm{correct}}}{N_{\mathrm{total}}}$$ (9)

where:

*N*_*correct*_ = number of correct inferences.

*N*_*total*_ = total number of inferences.

The scalpel tracking process involved two different sensors. A RealSense D415 camera set in RGB mode was used for the purpose of surgical tool localisation using SimPS-Net in real-time. This camera can operate in two different modes, namely stereo and depth mode. When used for depth measurements, the provided error is ±2% (Intel RealSense, 2023). However, in this experiment, the camera was set in stereo mode, and only the feed of one of the two lenses was utilised as the input to the network, effectively making the utilised camera a monocular system. Additionally, a stk300 optical tracking system from Atracsys (Switzerland) was employed to record the true 3D pose of the scalpel at each step, with an error of ±130 *μm* [(Atracsys, 2020).

Note, considering the errors accompanying the network position and orientation accuracy, two different thresholds were defined. A positional threshold was established at 9 mm, and an angular threshold was defined at 5° respectively. These values were defined by adding 50% of the accuracy errors to the actual values of position and orientation accuracy. While these thresholds suggest accepting some values with high positional deviation from the ground truth, especially compared to the accuracy of optical tracking solutions, it is important to ensure that a substantial amount of recorded points is gathered for the purpose of active constraint definition. During network deployment, the network inference was compared to the true pose obtained via optical tracking. The network pose results with an absolute difference lower than the position and orientation thresholds were accepted as correct inferences, with any other results being considered false. Moreover, to ensure the network could operate at a sufficient rate in real-time, it was deployed using a NVIDIA GeForce RTX 3070 and Tensorflow 2.0, achieving a frame rate of 4.5 frames per second.

### 2.4 Active constraint definition

**FIGURE 6:**
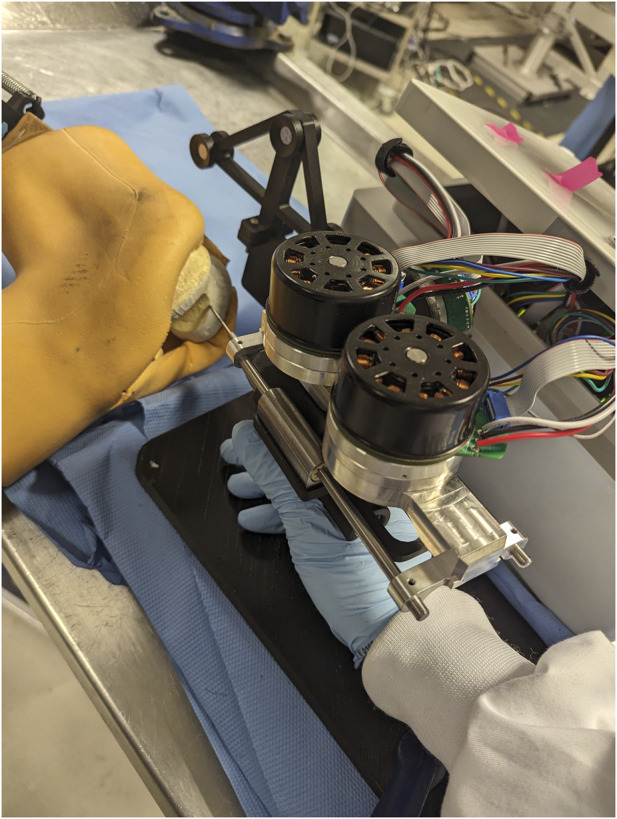
Experimental Setup of Robot and Phantom Knee: Presents the arrangement of the robot and the phantom knee in the laboratory, with a human operator manipulating the surgical tool to interact with the constraint.

The ability to track the 3D trajectory of a scalpel held by the operator can be used in the process of active constraint definition. The purpose of this part of the experiment is to demonstrate how a surgical robot operator can define an active constraint boundary intraoperatively, without the need to use any preoperative imaging methods. By expressing the trajectory of the tracked scalpel in 3D robot coordinates, the operator may define a constraint region that does not involve spatial registration with the operated geometry. Instead, the active constraint boundary can be defined “on-the-fly,” by only being localised in the robot workspace. To explore this proof of concept, along with the constraint enforcement demonstrated in the next section, a phantom knee joint was deployed in a cadaveric lab. Both the constraint definition and enforcement were undertaken by an operator familiar with both the localisation network as well as the Signature Robot. Note, the phantom knee was used solely for the purpose of better mimicking the conditions of a surgical operation, as well as better recreate a scene as similar to the dataset used for training of SimPS-Net as possible. No actual cutting of the phantom knee was performed in the active constraint experiments. The setup is shown in Figure 6, with the operator manipulating the robot tooltip but not actually performing any cuts. Note, a screen used for visualising the constraint definition process is not included in the image, but is accessible to the operator.

Prior to active constraint definition, a note needs to be made regarding frames of reference. Before recording the trajectory of the scalpel, the robot was rigidly fixed in place with respect to the RGB camera and the optical tracker. The RGB camera was also fixed with respect to the optical tracker. The robot was registered to the optical tracker, thus allowing for conversion between coordinate axes of the robot and the tracker. Additionally, via the same extrinsic calibration method previously reported (Souipas et al., 2023b), the camera 3D coordinates were also registered to the coordinate frame of the optical tracker. As a result, any inference made in the camera frame of reference could be expressed in the workspace of the robot.

The process of establishing an active constraint geometry involved the collection of the scalpel trajectory. The trajectory was then used to generate an area. The area was ultimately extruded to generate a 2.5D active constraint volume. This volume could then be used for testing constraint enforcement. The process of active constraint definition and enforcement constitutes a single test, and 10 tests were undertaken.

**FIGURE 7:**
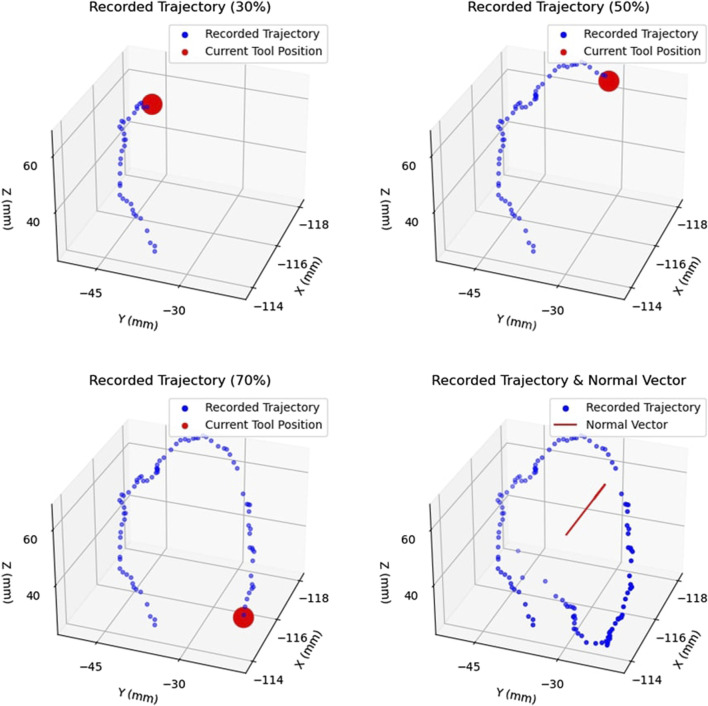
Example of Area Generation: Demonstrates the process of creating an area by recorded the trajectory of the tracked surgical tool. The trajectory is shown at 30%, 50%, and 70% completion, with the current tool position shown in red. The complete trajectory and the calculated normal vector, $\hat{n}$ , are also shown in the final subfigure. This area forms the basis of the generated volume in the next step.

To define the active constraint, the operator was required to generate a collection of non-coplanar points in 3D space using a scalpel, which the operator manipulated in space freely using their hand. Figure 7 demonstrates the process of producing an area. The subfigures demonstrate the evolution of the recorded trajectory for one experiment. Each accepted position of the scalpel was recorded at a fixed rate, shown in blue, with the current position of the scalpel tip shown in red. Once the operator decided to terminate the recording, the final point was connected to the initial point of the collected trajectory. This was achieved by first constructing the vector between these two points, and calculating the distance between them. Having done so, the obtained vector was “padded” with one point per 1 mm, thus effectively closing the generated area. Subsequently, principal component analysis (Kurita, 2020) was applied to the collection of recorded points, thus identifying the theoretically optimal plane, as well as the normal to the plane, $\hat{n}$ . The normal vector was placed at the centroid of all collected points. Upon obtaining $\hat{n}$ , the vector normal to the theoretical plane of the recorded scalpel trajectory, an area was constructed. This is demonstrated in the final subfigure of Figure 7, where the collection of points is shown in blue, with the normal vector, $\hat{n}$ , being shown in red.

**FIGURE 8:**
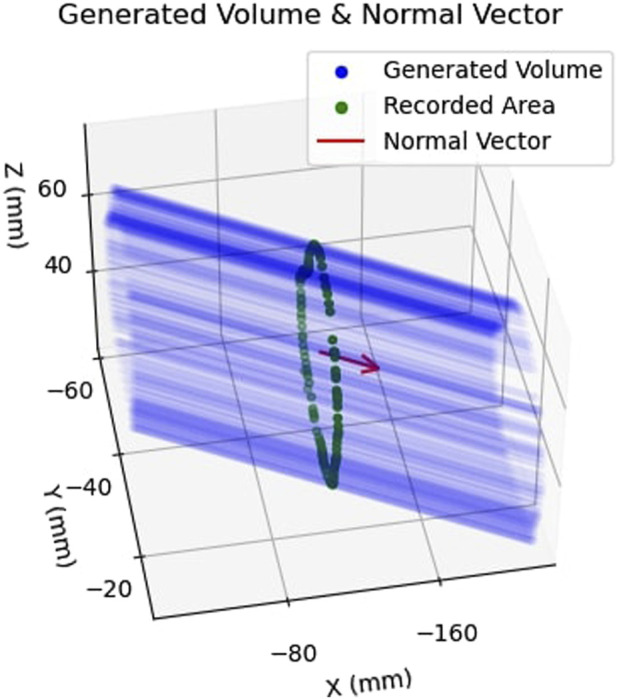
Example of Safety Volume Generation: Shows how the previously obtained area, shown in green, is extruded along the normal vector, $\hat{n}$ , shown in red to obtain the extruded safety volume, shown in blue.

Having defined an area in 3D camera space, the collected points were subsequently extruded in space parallel to the normal vector, $\hat{n}$ , by a fixed depth of 50 mm along each direction of the vector. As a result, a 2.5D extruded volume was generated. An example of this volume is illustrated in Figure 8, with the extruded volume shown in blue, the normal vector in red, and the area used as the basis of the constraint geometry shown in green. The volume, expressed in camera 3D coordinates, was used as the active constraint geometry for a single test. For each test, the constraint geometry was first defined, followed by the process of constraint enforcement. Note, upon defining the active constraint boundary in camera coordinates, the aforementioned registration results were utilised to ultimately express the boundary in robot 3D coordinates.

### 2.5 Active constraint enforcement

The final step of the proposed pipeline involves the enforcement of the active constraint on the Signature Robot. With the constraint having been defined in the previous section, two different constraint enforcement methods were tested. Firstly, a safe-zone was tested. In this constraint enforcement, an attractive force is applied on the robot tooltip upon penetration of the constraint boundary. The second enforcement method was a restricted-zone. In this mode, a repulsive force is applied on the robot tooltip as it is moved closer to the constraint boundary. Note that both these experiments shared a common constraint definition at each repetition.

#### 2.5.1 Safe-zone testing

With the constraint geometry established, and the robot rigidly fixed in space, the safe-zone constraint was tested. The human operator used their hand to manipulate the robot end-effector, starting within the safe-zone, and moving the tool in space. Each enforcement test was performed under two modes, once in freehand mode, where no constraint was applied, and once in haptic feedback mode, where haptic force feedback was provided. Furthermore, neither mode of operation allowed for visual feedback through the use of the digital twin.

**FIGURE 9:**
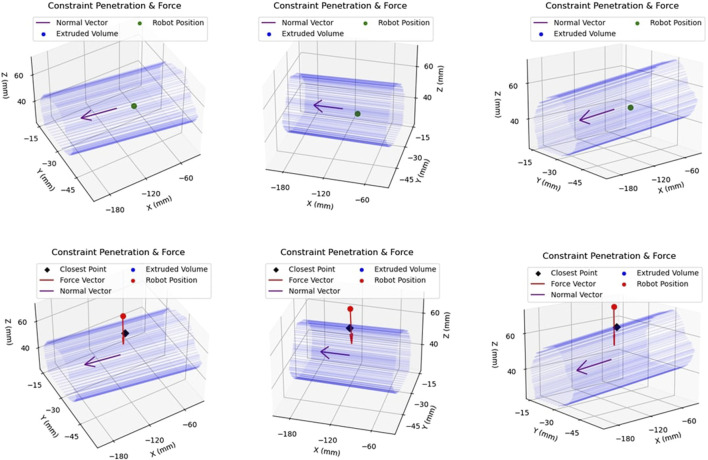
Example of Constraint Enforcement on Robot Tooltip: The top row of figures demonstrates the same location of the robot end-effector within the safety volume, hence depicted in green to illustrate that no haptic force is applied to the tooltip. The bottom row demonstrates another location of the robot, this time outside the constraint volume, along with the applied force vector in red.

While operating under haptic feedback mode, a translational force was applied to the operator’s hand by the robot in case of constraint boundary penetration. The purpose of this attractive force was returning the user back within the safe-zone. The force was calculated in the same manner as the plastic bone robot testing experiments of Section 2.1, with Equation 1 being used to calculate the force. However, since the generated constraint geometry was not continuous, the nearest point of the constraint boundary was instead identified. Subsequently, the distance, d, was defined as the distance between the robot tooltip and that identified point on the boundary. This is demonstrated in Figure 9. The top row of figures demonstrates motion within the constraint geometry, with the robot tooltip shown in green. The vector normal to the extruded area, $\hat{n}$ , is also shown. Note, the three figures of the top row demonstrate the same robot position from different viewing orientations. The bottom row of figures demonstrates the case of constraint penetration. The robot position, this time shown in red, is outside the active constraint geometry. The identified closest point across the boundary is shown in black. The distance between the two is used to calculate the force magnitude. The force vector, shown in red, is directed towards the normal vector of the 2.5D extruded volume, $\hat{n}$ , shown in purple.

The metrics calculated in the safe-zone experiment were identical to those collected in the plastic bone experiment cutting, outlined in Section 2.2. Volume metrics were not collected, since active constraint enforcement experiments did not involve cutting procedures. It should be pointed out that a threshold of 100 ms was imposed on the experiments, below which an exit was not taken into consideration. This was done to eliminate cases of very low deviations from the boundary (<0.1 mm) that were occasionally observed in some exits that lasted an insignificant amount of time, which in this case was defined as 100 ms.

#### 2.5.2 Restricted-zone testing

In restricted-zone testing, the robot tooltip was initially positioned outside the constraint boundary. The operator could use their hand to manipulate the robot end-effector, however, a repulsive translational force was applied to the hand of the operator. This force was tuned to fully prevent penetration of the constraint boundary. Equation 10 demonstrates the inverse power law used to calculate the translational repulsive force:

$$F=\frac{\psi }{d^{2}}\cdot \hat{r}$$ (10)

where:

*ψ* = force tuning constant.

*d* = distance between robot tooltip and nearest point across constraint boundary.

$\hat{r}$ = unit vector pointing away from constraint geometry normal axis.

Note, *ψ* is used for tuning the force magnitude, ensuring that the forces experienced by the operator are sensible. Furthermore, d is similar to the distance measured during safe-zone testing, and demonstrated in Figure 9. Lastly, $\hat{r}$ is the vector between the current robot position and the normal vector, $\hat{n}$ , but points away from the constraint boundary, thus ensuring the force is repulsive. The value of *ψ* was empirically set to 1.0 *kN* ⋅ *m*.

The inverse power law can lead to unsustainable forces as the distance from the boundary decreases, or even become unsolvable if the constraint boundary is reached. For that reason, a threshold of 1.3 *mm* was set. In case the end-effector reached a distance to the boundary lower than this, the distance was still locked to equal this value, thus generating a force plateau at small distances. With this distance threshold established, and the aforementioned value of *ψ*, Eq. 10 results to a maximum force of 59.2N, which is close to the maximum force that the linear axis of Signature Robot can sustain. A similar result could be achieved by increasing both the values of *ψ* and the distance threshold, however, since this is the first implementation of the restricted area experiments, the value of *ψ* was kept as straightforward as possible.

For all 10 repetitions, the operator attempted to penetrate the restricted-zone, with force and distance from the boundary being recorded. Similarly to the safe-zone testing, no visual feedback was available.

## 3 Results

A total of 4 volunteers were asked to perform plastic bone cutting experiments, undertaken for testing the Signature Robot platform. In addition, 10 repetitions were completed for the process of “on-the-fly” active constraint definition and enforcement testing. The following subsections list the results for these two different sets of tests.

### 3.1 Signature Robot testing

Four different modes of operation were explored by 4 volunteers in order to understand the capabilities of the current version of Signature Robot. Volunteers were asked to define the start of a cylinder on a plastic bone, and then attempt to cut out the defined cylindrical section. Across the participants, some important parameters, namely the average individual exit distance and duration, as well as the average of maximum exit distances and durations were measured, in accordance to the method outlined in Section 2.2. In addition, the ratio of correctly burred volume, i.e. volume within the constraint geometry, over total burred volume, was calculated. Table 3 lists the results across the four modes of operation, along with the relevant uncertainties. Note, all metrics are reported with an error of one standard deviation.

**TABLE 3:** Results of Plastic Bone Cutting Experiments: Demonstrates the results obtained using the Signature Robot under the four different modes of operation, along with the relevant errors (± standard deviation).

Test	Average individual exit distance (mm)	Average of maximum exit distances (mm)	Average individual exit duration (s)	Average of maximum exit durations	Inner/Total volume ratio (%)
Test 1 - Freehand	0.60 ± 0.06	3.23 ± 0.47	0.73 ± 0.28	2.76 ± 1.26	63.5 ± 23.8
Test 2 - Visual Feedback	0.33 ± 0.06	1.57 ± 0.42	0.40 ± 0.11	1.53 ± 0.97	91.4 ± 0.28
Test 3 - Haptic Feedback	0.28 ± 0.05	1.43 ± 0.44	0.55 ± 0.21	2.14 ± 0.74	90.4 ± 2.33
Test 4 - Combined Feedback	0.13 ± 0.02	0.57 ± 0.07	0.28 ± 0.04	0.70 ± 0.32	95.7 ± 1.64

For each test, *Average Individual Exit Distance* is the mean of all individual exit distances across all volunteers, the *Average of Maximum Exit Distances* are the mean of the maximum distance achieved by each volunteer, the *Average Individual Exit Duration* is the mean of all individual exit duration values across all volunteers, and the *Average Maximum Exit Durations* are the mean of the maximum duration achieved by each volunteer. Lastly, the final metric is ratio of *Inner/Total Volume Ratio*, calculated as the portion of volume removed from within the cylindrical constraint over the total volume removed during testing.

Furthermore, each participant filled out a NASA TLX form at the end of the tests, with the results being amalgamated and averaged out on Table 4. This standard form addresses several parameters that, while subjective based on the experience of each participant, may provide some insightful conclusions in terms of ease of operation. Such feedback can, in turn, be incorporated into future design improvements or additional features. The form was completed by each participant upon completing the plastic bone burring process in all four modes of operation. Note that the maximum value of the scale used was 20.

**TABLE 4:** NASA TLX Results: Lists the average response across all volunteers for the presented metrics.

Mental demand	Physical demand	Temporal demand	Performance	Effort	Frustration
4.7 ± 1.1	12.8 ± 1.8	2.7 ± 0.7	16.3 ± 1.6	6.0 ± 3.1	4.7 ± 2.6

A NASA TLX assessment form was filled by each volunteer upon completing all four experiments in order to explore the mental demand involved in visual feedback, the physical demand of manipulating the robot end-effector, the temporal demand of performing an experiment, the perceived performance, the effort required to complete a test and, finally, the frustration of each volunteer in operating the presented robotic platform. Note, standard deviation was used as the ucnertainty value for each metric. The scores are on a scale of 0–20.

### 3.2 Active constraint testing

Upon conducting 10 iterations for active constraint testing, the average network success rate was found to be 54.7% ± 5.2%. Following each active constraint definition output, the results of boundary interactions in safe-zone testing and restricted-zone testing were obtained. These results were then amalgamated for all iterations. Firstly, when examining the safe-zone experiments, metrics for both freehand operation and haptic feedback operation were collected, with the combined results presented in Table 5. The parameters of interest are calculated in a similar manner as those reported in Table 3. The reported errors are the standard deviations of each parameter.

**TABLE 5:** Results of Active Constraint Experiments: Demonstrates the results obtained using the combination of Signature Robot and the defined active constraint using tracked surgical tools by comparing freehand operation and haptic feedback operation (controlled).

Mode	Average individual exit distance (mm)	Average of maximum exit distances (mm)	Average individual exit duration (s)	Average of maximum exit durations
Freehand	5.83 ± 1.65	20.74 ± 6.78	1.32 ± 0.56	3.69 ± 1.97
Haptic Feedback	2.70 ± 0.37	9.58 ± 2.48	0.76 ± 0.11	2.03 ± 0.51

The values, along with the relevant errors (± standard deviation) are obtained by averaging relevant measurements across all 10 repetitions for each mode of operation. For each test, *Average Individual Exit Distance* is the mean of all individual exit distances across all repetitions, the *Average of Maximum Exit Distances* are the mean of the maximum distance measured, the *Average Individual Exit Duration* is the mean of all individual exit duration values, and the *Average Maximum Exit Durations* are the mean of the maximum duration achieved by in each repetition.

**FIGURE 10:**
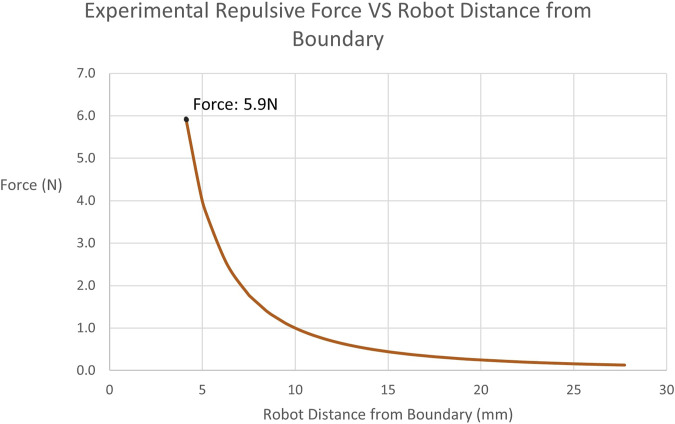
Experimentally Obtained Forces on Tooltip in restricted-zone Testing: Illustrates the recorded repulsive force values applied by the robot to the user when approaching the restricted-zone constraint region during testing. The maximum force achieved is 5.9N at a distance of 4.11 mm.

Subsequently, the restricted-zone tests mostly focused on proving that the robot can be completely prevented from entering the defined region, when the operator does not actively attempt to penetrate the constraint by exerting forces higher than what they normally would apply. Indeed, throughout all iterations of the test, not a single boundary penetration was observed. Figure 10 demonstrates the force exerted during one of the experiments as a function of the distance of the robot from the active constraint boundary.

## 4 Discussion

The main purposes of this research were to both demonstrate how the Signature Robot was further improved from previous iterations, as well as establish an “on-the-fly” active constraint definition method, which could ultimately be enforced through the Signature Robot. The constraint geometry generation technique presented offers a noteworthy level of versatility, as it does not involve the use of any optical tracking, whilst also allowing operators to seamlessly produce a constraint boundary intraoperatively, by employing standard surgical tools. This latter point suggests that surgeons need not familiarise themselves with unknown tools in order to define a constraint. Through the measurements obtained during the two separate experiments, namely the plastic bone cutting experiment and the active constraint experiment, it is possible to understand the capabilities of all systems presented throughout this paper, as well as understand limitations of the current techniques which will need to be addressed in the future.

### 4.1 Signature Robot

The Signature Robot was developed as a collaborative, hands-on system for orthopaedic surgery. Throughout the design of this platform, the aim of miniaturisation was maintained in order to minimise the footprint occupied in the operating room. Furthermore, the system is capable of providing haptic feedback to the operator. With the presented version employing a direct drive for each of the three degrees-of-freedom, the system is indeed capable of providing a sufficient force for human bone grinding and cutting. The static, cylindrical active constraint tested during plastic bone cutting tests is a promising starting point, and the highly tunable force algorithm presented in Eq. 1 suggests that with further qualitative testing, an optimal value of elastic and damping coefficients can be identified.

By observing Table 3, it is possible to draw some general conclusions. Specifically, the metrics of freehand operation are principally higher, both in terms of exit duration but also in terms of distance from the boundary. In the absence of feedback, the volunteers were dependant solely on spatial awareness, and any deviation from the defined constraint geometry could not be limited. This is further underlined in the metric of inner volume over total volume. This ratio indicates the amount of correctly removed volume during operation. A lower percentage means that a higher portion of volume was incorrectly removed. The ratio of 63.5% exhibited in freehand operation, along with the significantly high error of 23.8%, suggests that a portion of the volunteers estimated the spatial position of the constraint boundary incorrectly, thus burring out a significant volume of the plastic bone specimen falsely.

Some interesting findings, however, are observed when comparing visual feedback and haptic feedback. Specifically, the results of these two modes of operation are highly comparable. Haptic feedback leads to marginally lower constraint boundary deviation distances, exhibiting an average distance of 0.28 mm, compared to the 0.33 mm value under visual feedback. Conversely, deviations from the boundary under visual feedback last less than exits under haptic feedback. One explanation for this observation could be that in visual feedback operation, constraint boundary penetration is presented in a binary manner. This is demonstrated in Figure 2, where upon exiting the constraint boundary, the colour instantly changes from green to red. This form of feedback may trigger a swifter reaction from operators, without necessarily minimising the distance from the boundary. Conversely, haptic feedback response is highly dependent on the distance from the boundary. Lower distances are perceived more difficultly, thus suggesting that an exit duration may last longer, but the robot end-effector will remain closer to the boundary during the exit. This observation could suggest that volunteers developed a level of confidence on their operation of the robot, thus not responding to low force levels since higher exit distances would be prevented. A more realistic explanation, however, is that the low force levels were not perceived. One of the limitations of this study is the low number of participants, which prevents any statistically significant observations. To address the imperceivable force values, it is possible to further increase the elastic constants of the viscoelastic force model, hence leading to a stiffer force being applied upon penetrating the constraint boundary. However, a balance is needed between constraint stiffness and freedom of operation, since in reality, surgeons need to remain in control, instead of being completely restricted by the robotic platform.

A final observation is that upon combining visual and haptic feedback, the results of both exit distance and duration fall significantly. Under combined feedback, the average of maximum exit distances noted across the four volunteers remained below 1 mm, with the average of maximum exit durations also remaining below 1s. In addition, in the presence of combined feedback, the volume burred during operation was almost entirely removed from within the constraint, at a ratio of 95.7%. These results of combined feedback suggest that, even though haptic feedback can be used to reduce the distances from the boundary upon penetration, and visual feedback can reduce the respective duration, it is the combined feedback that exhibits the highest reduction of both these values, and therefore the combination of both yields more benefits than the application of individual forms of feedback. However, another limitation of this study was the use of plastic bone phantoms for testing. Realistically, in human bone specimens, the involved forces would be more significant. Another concern is that upon penetrating the cortical bone and reaching the trabecular bone, the operated tissue becomes weaker, and the operator may accidentally apply a significant force to cut through the trabecular bone. Cases like these, where the resistive force abruptly changes in operation, need to be further explored in order to fully understand the benefits of visual, haptic, and combined feedback. Ultimately, it is not possible to apply the findings from cutting plastic bone to human bones. Nevertheless, this study was a promising initial demonstration of feedback performances, but also a satisfying display of a proof-of-concept robotic system with haptic and visual feedback capabilities.

In addition to the numerical results discussed above, the volunteers were also asked to complete a TLX form, with the amalgamated results shown in Table 4. With a brief examination, one may conclude that the main shortcoming of this platform is the physical demand of operation. Indeed, the direct drive design suffers from significant weight, weighing 1.9 kg. The weight of the mechanism is also entirely supported by the hand of the operator, which in turn causes increased physical demand. This finding suggests that the robot would greatly benefit from the implementation of a gravity compensation algorithm, thus alleviating the load on the operator during surgery.

Examining some other metrics, it is interesting to note that under the combination of visual and haptic feedback, the volunteers still reported a low mental demand value of 4.7. This suggests that the visual feedback, even though implemented via a straightforward colour change and a vector arrow presentation of the force, provides satisfactory results while not mentally straining the operators. However, the limited sample size of 4 volunteers needs to be expanded in order to draw more significant conclusions.

### 4.2 Active constraint definition

With regard to the constraint definition and enforcement experiments, the relevant results also allow for some generalisations. Firstly, as mentioned in Section 3.2, a real-time success rate of 54.7% was achieved for the employed 3D localisation network, SimPS-Net. The employment of SimPS-Net for the purpose of tracking a standard surgical tool in space and subsequently defining an active constraint boundary based on the recorded trajectory presents a highly versatile, intraoperative constraint definition technique. Moreover, no spatial registration to the patient anatomy was required, thus reducing the time required to generate the constraint boundary, while also not requiring surgeon supervision of external devices to define a new active constraint.

It should be noted, however, that as explained in Section 2.3, the values accepted as correct inferences were within 9 mm and 5° of the true location of the scalpel in position and orientation respectively. These thresholds are too generous for orthoapedic surgery applications. Nevertheless, such thresholds were necessary in order to account for the fact that this experiment took place using a phantom knee joint. This specimen differs from the dataset used for the training and testing of the network, which involved data collected from a cadaveric knee specimen (Souipas et al., 2023b). Ultimately, while the thresholds used were higher than the errors reported by the network, the real-time implementation of the localisation network still provides some promising results.

Another limitation of the presented implementation is the incorporation of optical tracking to convert the constraint boundary from camera 3D coordinates to robot 3D coordinates. This requirement prevents the pipeline presented in this paper from being completely independent of optical tracking, which would be a significant step towards an even more versatile and deployable active constraint definition technique.

### 4.3 Active constraint enforcement

With the constraint boundary having been generated, the Signature Robot was tested under safe-zone and restricted-zone constraint enforcement. In the former, the end-effector was freely manipulated within the constraint region, and attractive forces were only applied upon exiting the constraint boundary, thus guiding the operator back within the constraint geometry. The restricted-zone testing was enforced in a separate test, with the purpose of preventing the robot tooltip from entering the generated active constraint boundary.

#### 4.3.1 Safe-zone testing

Under safe-zone testing, the user was allowed to explore the generated constraint geometry initially in freehand mode, where no haptic force feedback was involved. Subsequently, the operator tested the constraint in “controlled” mode, meaning operation under haptic force feedback. The results for these two modes of operation are demonstrated on Table 5. Similarly to the plastic bone cutting experiments, in the case of freehand operation, the user solely depended on spatial awareness, as visual feedback was absent. By examining the results, it can be stated that the presence of haptic force feedback leads to an improved performance, both in terms of exit distances, but also in terms of duration of exits.

The results demonstrate significant reductions in both exit distance metrics but also exit duration metrics. Specifically, in the absence of haptic force feedback, the average distance of the robot from the constraint boundary increased by 116%, with a similar rise in the average of maximum distances by 117%. Similarly, the average duration of individual exits increased by 74%, with the average of maximum exit duration rising by 82%. In contrast, controlled operation ensured the mean duration of each exit remained below one second, thus demonstrating the presence of haptic feedback greatly reduces the exit duration. These findings further underline that haptic feedback allows the operator to depend on more robust information than their own spatial awareness, which is prone to compounding errors due to incorrect initial offsets of the end-effector.

Comparing the haptic feedback results in Table 5 with those listed under haptic feedback operation in Table 3 for plastic bone cutting, it is noted that exit distance metrics are significantly higher under safe-zone testing. Specifically, the average individual exit distance in safe-zone testing is 2.70 mm, which is vastly higher than its plastic bone counterpart of 0.28 mm. The explanation behind this discrepancy is that even though both experiments shared an equal value for the elastic constants in Eq. 1, the volunteers in plastic bone testing had to overcome the resistance posed during cutting of the bone phantom specimens. This force provided an additional resistance to the viscoelastic constraint enforcement, thus allowing volunteers to perceive the haptic force feedback and react to it before reaching significant boundary penetration distances. Conversely, the safe-zone testing was undertaken in unconstrained space, with no resistance imposed to the operator’s hand other than the haptic feedback force. Therefore, in the case of safe-zone testing, greater distances were reached prior to any reaction by the operator. This is further underlined by the duration metrics. Specifically, the average individual exit duration and the average of maximum exit durations are comparable across the two experiments. This observation could verify the fact that operators take the same time to respond to haptic feedback, however, in the absence of cutting forces, the operator is allowed to penetrate the constraint boundary at higher distances prior to returning within the constraint geometry.

A way to address this discrepancy would be to use stiffer elastic constant values in safe-zone testing, which could account for the absent resistive force of cutting through plastic bone specimens. A more robust solution, however, would be to repeat this experiment as a cutting experiment. In the safe-zone experiment presented in this paper, the operator was allowed to move unrestricted in space. An improvement would be to perform cutting operations in either phantom or human bone specimens, thus better exploring the constraint enforcement process.

#### 4.3.2 Restricted-zone testing

Examining the restricted-zone testing, the main result is that across all 10 repetitions, no successful penetration of the constraint boundary was achieved. As mentioned, a force plateau was enforced, thus creating a maximum cap on the possible force. However, the operator never reached the threshold of 1.3 mm established in Section 2.5.2, since the force exponentially increased at a distance of 5 mm from the boundary. This was achieved by tuning the value of *ψ* in Eq. 10. As expected with a power law, the force increased at a high rate the closer the robot tooltip was positioned to the boundary. Therefore, this experiment demonstrates that a restricted-zone constraint can be imposed on the Signature Robot and successfully prevent boundary penetration. A limitation of this experiment is that the operator was restricted to forces applied through finger manipulation of the robot end-effector. Instead, in a future iteration of this experiment, a process for exploring higher levels of repulsive force should be implemented. Regardless of this limitation, however, the result of complete prevention of boundary penetration is very promising, and with Eq. 10 being very tunable, different force values could be explored.

### 4.4 Conclusion & future work

Throughout this paper, both the assembly of a collaborative, hands-on, surgical robot and the definition and enforcement of an “on-the-fly” active constraint were presented and analysed, with the constraint ultimately being enforced on the Signature Robot.

In terms of robot development, it was shown that the haptic capabilities of the system can improve the performance in operation, even when it comes to untrained volunteers. Furthermore, the results when operating on plastic bone can be enhanced through the fusion of haptic and visual feedback, thus resulting in submillimeter boundary penetration distances from a constraint boundary. Ultimately, this novel platform is a promising starting point that addresses the absence of miniaturised orthopaedic robots with haptic feedback capabilities. The Signature Robot could, however, benefit from the implementation of gravity compensation, which will significantly lower the physical demand of operating the robot. Furthermore, the establishment of a calibration method that would allow each operator to identify the optimal elastic and damping coefficients could improve the performance of haptic feedback operation, while still ensuring each operator is satisfied with the chosen values.

Regarding the constraint definition and enforcement, it was demonstrated that a real-time, monocular, RGB camera-based network capable of surgical tool localisation, SimPS-Net can be used for “on-the-fly” constraint definition. A constraint geometry was produced using this network and a standard scalpel, thus allowing the operator to define a constraint geometry intraoperatively, without the need to spatially register the generated constraint on the patient anatomy before registering it to the robotic platform itself. This technique could further improve by establishing a registration technique between robot and camera, allowing for the registration of the generated constraint geometry directly to the robot workspace, without the need of optical tracking. In doing so, the proposed active constraint definition solution could offer even more versatility and be more deployable than its current conception. In doing so, extrinsic calibration between the camera and the optical tracker could be avoided, thus further reducing the time required to define a constraint. In addition, this method can eliminate the need to redesign standard surgical tools in order to accommodate trackable bodies.

Examining the constraint enforcement process, the benefits offered by the introduction of haptic feedback were confirmed under safe-zone testing. In addition, complete prevention of constraint boundary penetration was achieved through the established pipeline in restricted-zone testing. Both these experiments demonstrated that a constraint can be successfully imposed on a robotic platform after being generated “on-the-fly” using the presented technique. Therefore, patient tissue safety can be further enhanced by integrating this technique along the robotic surgery workflow. A future repetition of these experiments will aim to optimise the constants used in force calculation equations, providing sufficient stiffness to minimise boundary penetration distances. In addition, the experiment should be performed with the robot used for cutting or burring of a specimen, to better demonstrate the benefits of the proposed constraint definition and enforcement pipeline solution.

A final point to make is the need to implement a more robust algorithm for constraint enforcement, both in terms of plastic bone testing and also safe-zone testing. The viscoelastic model stores energy, and can lead to patient tissue injury upon releasing the robot end-effector unintentionally. More robust algorithms, such as the frictional model (Bowyer and Ferdinando Rodriguez, 2014), have been proposed in the literature, and therefore integrating them in the haptic model of Signature Robot would make the entire system safer and easier to manipulate.

## Data Availability

The raw data supporting the conclusion of this article will be made available by the authors, without undue reservation.
